# The use of composite time trade-off and discrete choice experiment methods for the valuation of the Short Warwick-Edinburgh Mental Well-being Scale (SWEMWBS): a think-aloud study

**DOI:** 10.1007/s11136-022-03123-0

**Published:** 2022-03-24

**Authors:** Hei Hang Edmund Yiu, Hareth Al-Janabi, Sarah Stewart-Brown, Stavros Petrou, Jason Madan

**Affiliations:** 1 Clinical Trials Unit, Warwick Medical School, University of Warwick, Coventry, UK; 2 College of Medical and Dental Sciences, Institute of Applied Health Research, University of Birmingham, Edgbaston, Birmingham, UK; 3 Division of Health Sciences, Warwick Medical School, University of Warwick, Coventry, UK; 4 Nuffield Department of Primary Care Health Sciences, University of Oxford, Woodstock Road, Oxford, UK

**Keywords:** SWEMWBS, Preference elicitation, Think-aloud, Cognitive interview, Composite time trade-off, Discrete Choice experiment

## Abstract

**Purpose:**

To identify patterns and problems in completing composite time trade-off (C-TTO) and discrete choice experiment (DCE) exercises for the valuation of the Short Warwick-Edinburgh Mental Well-being Scale (SWEMWBS) to inform the optimisation of a valuation protocol.

**Methods:**

Fourteen cognitive interviews were conducted in the UK using concurrent and retrospective think-aloud and probing techniques. Each participant completed 8 C-TTO tasks and 8 DCE tasks within a computer-assisted personal interview setting. Verbal information was transcribed verbatim. Axial coding and thematic analysis were used to organise the qualitative data and identify patterns and problems with the completion of tasks.

**Results:**

While participants found the tasks generally manageable, five broad themes emerged to explain and optimise the response to the tasks. (1) Format and structure: attention to the design of practice examples, instructions, and layout were needed. (2) Items and levels: underlying relationships were discovered across different combinations of levels of SWEMWBS items. (3) Decision heuristics: participants engaged in diverse strategies to assist trade-off decisions. (4) Valuation feasibility: certain states were difficult to imagine, compare and quantify. (5) Valuation outcome: the data quality was affected by participants’ discriminatory ability across states and their time trade-off decisions.

**Conclusion:**

The interviews contributed insights regarding the robustness of the proposed methods. The application of C-TTO and DCE valuation techniques was practical and suitable for capturing individual attitudes towards different mental well-being scenarios. A modified protocol informed by the results is being tested in a larger sample across the UK.

**Supplementary Information:**

The online version contains supplementary material available at 10.1007/s11136-022-03123-0.

## Plain English summary

Governments funding health services often use research developed to value the effects of healthcare services on illness. To extend this to public services other than healthcare services, we need to understand how people value different aspects of well-being.

This study tests approaches developed by economists for valuing health to see how well they apply to valuing mental well-being improvements measured with a reliable, valid and widely used scale – the Short Warwick-Edinburgh Mental Well-being Scale (SWEMWBS).

Valuation tasks were designed on a digital platform based on methods used for valuing a measure very widely used in healthcare service evaluation (the EQ-5D-5L) and mental well-being profiles derived from SWEMWBS. Participants were asked to express their feelings and thoughts while completing the valuation exercise; these were recorded and analysed using qualitative methods.

Despite challenges identified by participants in understanding some elements of the valuation tasks, all participants managed to complete the tasks using diverse strategies. Participants thought that the tasks were beneficial and allowed reflection on their lives and personal preferences. A practical finding was that techniques developed to value health are also suitable for capturing individual valuations of different mental well-being profiles. These findings will be further tested and applied in future mental well-being valuation studies, to identify the best approach for valuing improvements in mental well-being that result from public services.

## Introduction

The quality-adjusted life year (QALY) is an outcome measure used to inform cost-utility analyses of healthcare interventions. However, many generic preference-based measures (e.g. EQ-5D-5L) used to derive health utility values for QALY estimation are subject to limitations as their descriptive systems focus mainly on physical dimensions of health, without sufficiently capturing other aspects of mental health [1–3]. Mental well-being (MWB) has been shown to be related to many aspects of morbidity, mortality and community outcomes [4–13]. As a good stage of life is more than the absence of physical problems, an alternative outcome measure named the “Mental Well-being Adjusted Life Year (MWALY)” has been postulated as an approach to capturing benefits of interventions related to broader aspects of well-being [14]. The SWEMWBS (reported in Online Resource 1) is a seven-item measure of MWB with five response levels from “none of the time” to “all of the time”. The items are positively worded to access hedonic and eudaimonic perspectives of well-being. The total scores range from 7 to 35 where higher scores indicate better MWB. Policymakers in Scotland, Wales and England use this questionnaire for monitoring population MWB [15–17]. It is psychometrically validated and widely recognised across diverse populations in the UK [18–20]. There is currently no generic preference-based MWB instrument for the economic evaluations of interventions that affect MWB. Therefore, identifying appropriate valuation protocols for measures of MWB is an important step towards the development of preference-based value sets.

A cognitive testing method is required to explore the validity of the valuation protocols. A common interviewing technique to understand the feelings and thoughts of information processing is the cognitive (or think-aloud) interview [21]. Cognitive interviews in health economics have tended to focus on identification of errors within questionnaire design [22–24], but they have also been applied in health preference valuation studies. Goodwin et al.[25] investigated the reasons for discrepancies between the TTO values derived by the general public and patients with multiple sclerosis. Respondents thought aloud their primary appraisal, secondary appraisal, and response process. A content analysis was used to understand the factors affecting respondents’ interpretations, judgements and trading criteria for the health states. Ryan et al.[26] applied think-aloud techniques to understand the cognitive process of completing DCE tasks related to choices for bowel cancer screening. Respondents’ potential violations of completeness, monotonicity and continuity axioms of utility were investigated. Results from qualitative research were used to complement quantitative data to explain seemingly irrational responses. Spencer [27] applied think-aloud techniques to analyse the completion of different variants of TTO for valuing EQ-5D-3L health states. The results were subsequently used to test the idea of procedural invariance. Although these studies provide some insights into the valuation techniques, we know little about the application of health state valuation techniques into the valuation of MWB. This study, therefore, aims to investigate the cognitive process of completing composite time trade-off (C-TTO) and discrete choice experiment (DCE) exercises for the valuation of the SWEMWBS to inform the optimisation of a valuation protocol.

## Methods

**Fig. 1:**
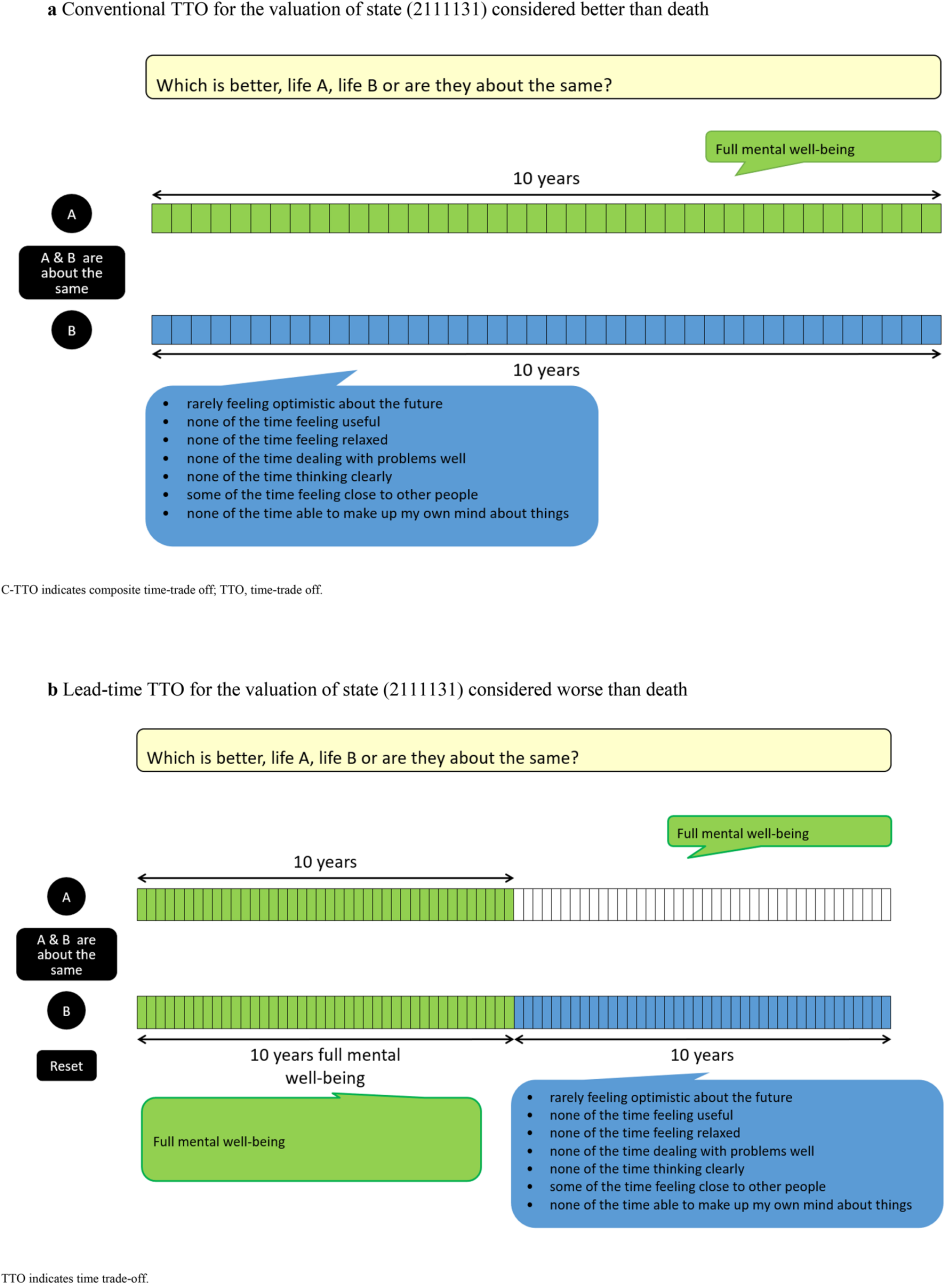
The C-TTO tasks

**Fig. 2:**
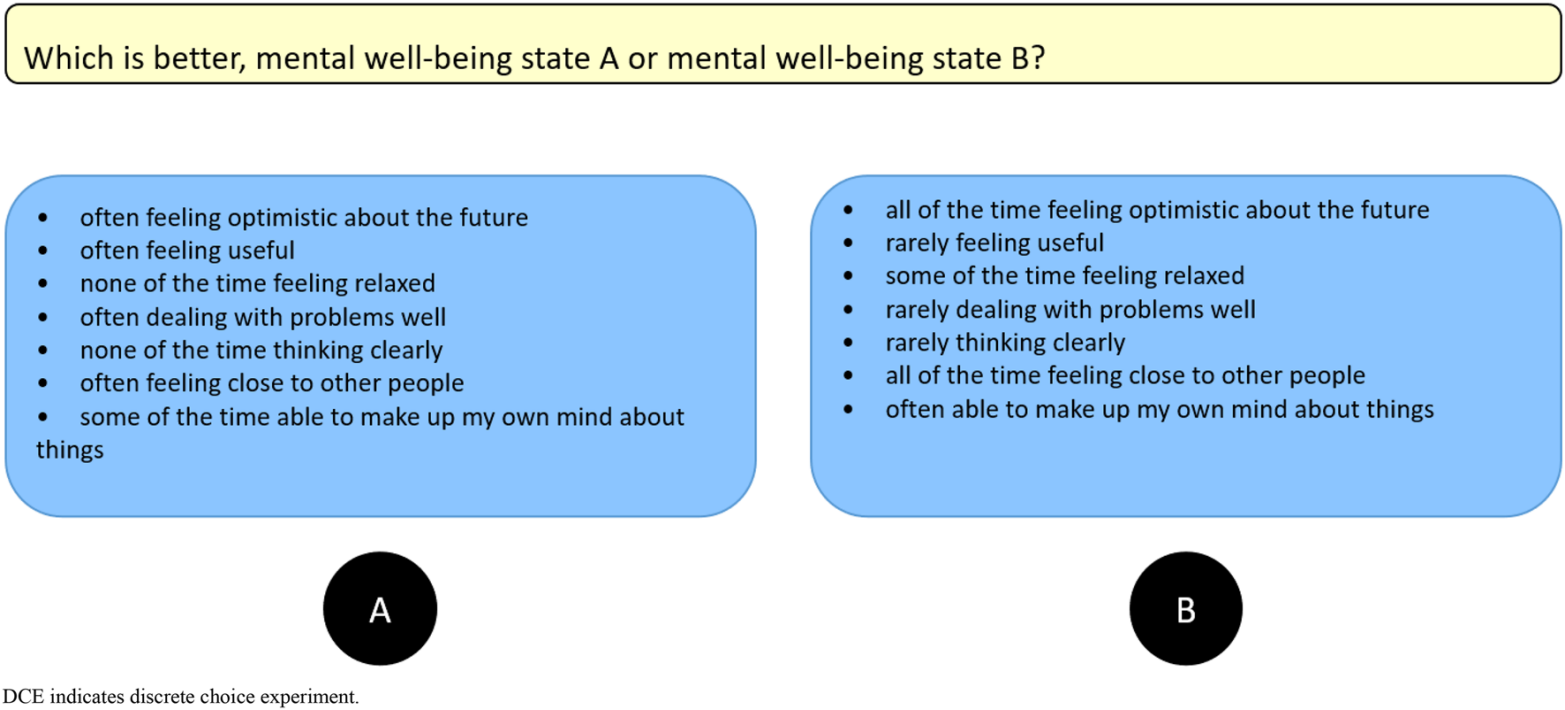
A pairwise DCE with forced choice

Face-to-face cognitive interviews were conducted to investigate the completion processes of the C-TTO (i.e. conventional TTO for the valuation of MWB states considered better than death and a lead-time TTO for states considered worse than death) and DCE exercises (examples shown in Figs. 1 and 2). Participants were asked to think aloud during and after the tasks within a computer-assisted personal interview setting.

A snowball convenience sample of the Warwickshire and West Midlands population in the UK aged 18 or above was recruited. The main source was university staff and students identified through personal networks. Motivated by the principle for specifying data saturation proposed by Francis et al.[28], the initial sample size was set at eight. The interviewer (HHEY) continued to recognise different themes of shared beliefs and the stopping point was applied when there were no new informative ideas identified for three consecutive interviews beyond the eighth interview.

### Experimental design for the selection of SWEMWBS states

For the DCE, assisted by the software Ngene, a D-efficient design with zero prior parameter values was used to systematically generate 32 DCE pairs, which were then randomly allocated into four blocks [29]. Each participant was asked to value one block, consisting of eight choice tasks. For the C-TTO, a blocked design was used. The lowest MWB state (1,111,111) and one of the states close to full MWB (FMWB) (4555555, 5455555, 5545555, 5554555, 5555455, 5555545 and 5555554) were included as two compulsory states within each block. Also, six additional states generated using the “AlgDesign” package in R were randomly allocated to each block. A level-balance criterion constructed by the EuroQol group was applied within each subset to check the number of appearances of each level-domain combination [30]. The best subset was randomly and evenly divided into seven blocks. Each participant was required to value one block.

### Valuation platform

The EQ-VT 2.1 was the most up-to-date platform with a strict quality control process developed by the EuroQol Group for recording C-TTO and DCE responses [31]. The EuroQol Portable Valuation Technology (EQ-PVT), a replica of the EQ-VT 2.1, was used throughout the interview and participants completed tasks displayed on the interviewer’s laptop.

### Interview process

**Fig. 3:**
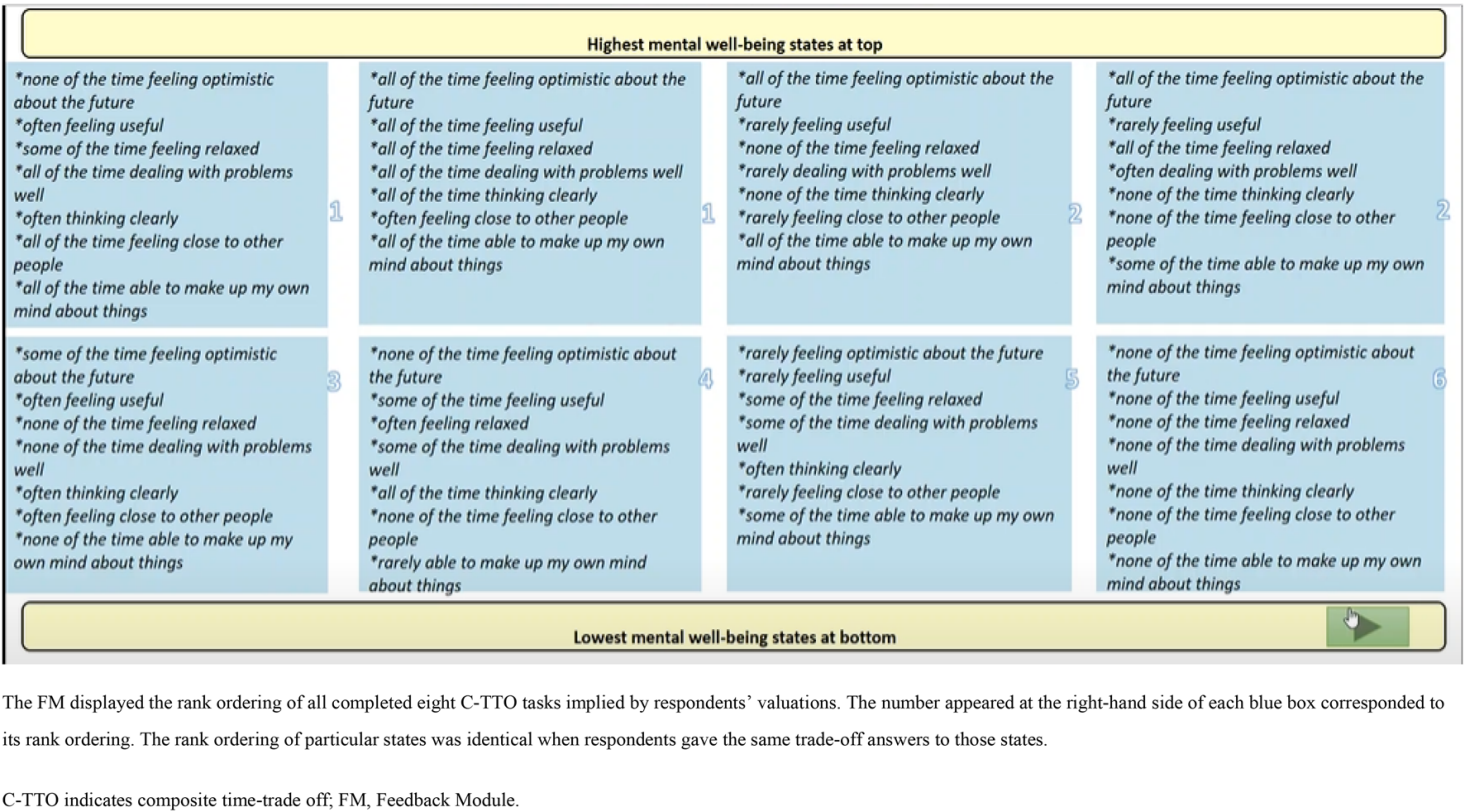
A visual presentation of the C-TTO FM

All interviews were audio recorded. Respondents were interviewed in their homes or at the university campus with the following procedure:

1. The interviewer introduced the study purpose.
2. The participant signed a participation consent form.
3. The participant completed the SWEMWBS in the Qualtrics survey tool describing their own MWB, followed by demographic questions. A think-aloud warm-up exercise involving ‘window counting’ was provided to the participant [32].
4. The C-TTO exercise: The participant was guided through examples of MWB states brought about by being regularly rejected following job applications. With reference to the EQ-VT 2.1, dynamic questions were added after the first practice example to allow interviewees to become familiar with another evaluation space [31]. Similarly, for the valuation of SWEMWBS, dynamic questions regarding the assessment of which state is better (i.e. being accepted for the most ideal job) and worse (i.e. regularly being rejected following job applications, and constantly suffering a poor relationship with friends) than the previous examples were asked for valuation. After these, three practice SWEMWBS states were provided: high (4554545), low (2111131) and intermediate (4212354) MWB states. Next, the participant completed the eight valuation tasks. To reduce recall bias, during the process of completing the first *three* tasks, each participant was asked to think-aloud everything that came to mind concurrently. To save time and reduce the respondent’s fatigue, each participant was asked to think-aloud retrospectively only after completing all *five* remaining tasks. Probing questions (Table 1) were used to complement the concurrent and retrospective cognitive process if they remained inactive.
  Finally, the rank ordering inferred by valuations was displayed in the Feedback Module (FM) (Fig. 3). Each participant was asked to flag any disagreements or inconsistencies with the results but was not asked to alter the problematic valuations. Some remaining debriefing questions (Table 2) were also asked if they were previously unaddressed.
5. The DCE exercise: The paired comparisons and the left–right order of each set of two states were randomised using the EQ-PVT platform. Concurrent think-aloud and retrospective think-aloud were applied to the completions of the first *three* and remaining *five* tasks, respectively. These were supplemented by probing questions (Table 1). Some remaining debriefing questions (Table 3 and 4) were also asked.

**Table 1:** Examples of probing questions during the think-aloud process for the C-TTO/DCE tasks

*“Could you tell me more about how easy/difficult completing this time trade-off task was?”*
*“You told me that you felt confused about determining the indifferent point for some of these 8 trade-off tasks/choosing between this pair of mental well-being profiles, could you tell me more about it?”*
*“What thoughts came to mind when you were making trade-offs between different mental well-being states/making a choice between this pair of mental well-being profile?”*

**Table 2:** Follow-up debriefing questions if they were not addressed within the think-aloud process of the C-TTO tasks

*“Were the practice tasks useful for you? How?”*
*“Did you think the instructions for the practice tasks clear for you?”*
*“Could you summarise the factors were you considering when deciding the indifferent point?”*
*“Did you find the number of valuation tasks (i.e. 8 trade-off tasks) manageable for you?”*
*“Could you tell me how easy/how difficult of completing these 8 valuation tasks were in general?*
*“Was the feedback slide useful for you?”*

**Table 3:** Follow-up debriefing questions if they were not addressed within the think-aloud process of the DCE tasks

*“Could you summarise the factors were you considering when deciding the most preferred option between pairs of mental well-being profile?”*
*“Did you find the number of valuation tasks (i.e. 8 tasks) manageable for you?”*
*“Could you tell me how easy/how difficult of completing these 8 valuation tasks were in general?”*

**Table 4:** Overall debriefing questions for both parts of the interview if they were not addressed within the think-aloud process

*“Did you think the first part of the interview (i.e. to make trade-off between choices of imaginable life) is easier or more difficult than the second part of the interview (i.e. to look at pairs of mental well-being profiles and choose the one you prefer)? Or did you feel roughly the same for both parts? Were they still manageable for you?”*
*“Was the total number of valuation tasks in this interview (i.e. 8 trade-off tasks and 8 choice tasks between pairs of mental well-being profile) manageable to you?”*
*“Would you prefer to have both parts of the interview or would you prefer only either one of them?”*
*“Do you have any final overall feedback or comments of this interview?”*

### Data analysis

After all interviews, verbal information was transcribed verbatim. Thematic analysis was used to analyse data collected by the concurrent and retrospective think-aloud techniques [33, 34]. First, open coding for the first four transcripts was performed by the first rater (HHEY) to identify task completion issues within the text. Coding was discussed and refined with a second rater (HA). With reference to the open coding for the first four transcripts and the field notes for the remaining transcripts, a coding tree for axial coding was then constructed by the first rater. Next, the axial coding framework was applied to code two informative transcripts by the first rater [35–37]. The second rater coded one of these transcripts and a third rater (JM) coded both transcripts. Upon completion of independent coding for the two transcripts, coding differences were discussed to enhance the consistency and reliability of the coding methods. A more robust version of the coding framework was developed after incorporating feedback raised by the raters. This was applied to code the remaining transcripts by the first rater. Nvivo was used for tagging and labelling potential codes. A codebook to describe the meaning of codes and a descriptive account to re-categorise the coding materials for generating higher-order themes were produced. An explanatory account was finally produced to selectively include quotes for the codes under each higher-order theme [35].

## Results

Fourteen interviews were conducted between 11th February and 18th March 2020. The interview time was ~ 60–75 min per participant. Table 5 describes the characteristics of participants. Participants highlighted the strengths and limitations of applying the valuation protocol and the completion process. Five broad themes were generated following analyses of the verbal text.

**Table 5:** Demographic characteristics of 14 participants

Characteristics	Number of participants
*Gender*	
Male	5
Female	9
*Age*	
18–30	3
31–40	5
41–50	2
51–60	2
> 60	2
*Highest education level attained*	
GCSE	1
O-Level	2
A-Level	2
Undergraduate	4
Postgraduate	
Master	2
PhD	3
*Ethnicity*	
White	12
Asian/Asian British	1
Arab	1
*Occupation*	
Administrator/Manager/Coordinator	6
Researcher	3
Student	1
Cleaner	1
Retired	3
*SWEMWBS score*	
25 or less	2
26–30	10
31–35	2
Mean score	27.64

SWEMWBS indicates Short Warwick-Edinburgh Mental Well-being Scale

### Theme 1: Format and structure

Participants appreciated the well-organised computer setting of the EQ-PVT platform and the automatic allocation of states. However, there were areas for improving the content of the tasks.

#### Inappropriate examples

Despite most participants understanding the C-TTO practice scenarios, two participants pointed to the irrelevance of the job searching example as they were not current job seekers.

“This is a really tough one… because I'm 67 and I don't really care about job applications”. (Female, 67)

#### Confusion on scenario completion

Two main sources of response errors for the C-TTO process were identified. (1) Mistakenly clicking the non-preferred option: five participants were confused about the transformation of their own preferences to appropriate clicks in tasks. (2) Failure to adjust length of life properly: participants sometimes had an indifference point between life A and life B in mind at first glance, but they struggled with the step-by-step procedure to reach that point.

“The scale is portrayed in a manner that my mind doesn't work. I find it quite strange to… delete and workup to equate a matching valuation”. (Male, 32)

The DCE exercise simply required participants to click on the preferred option between two scenarios and no option selection problems were identified.

#### Improvement of presentation layout

Two participants suggested the inclusion of pictures or colours instead of sole plain texts within the C-TTO FM, to enhance the differentiation of the eight MWB states with their corresponding attribute levels. In addition, nine participants disagreed with some of their own rank orderings of the eight completed C-TTO tasks. Although participants unanimously acknowledged the importance of reviewing their valuation answers, five participants suggested the possibility for allowing swapping of states after indicating disagreements.

### Theme 2: Items and levels

#### Contradiction in levels

Eight participants identified non-intuitive combinations of levels of items presented within states. This was a stumbling block to participants’ comprehension and imagination.

“often deal with problems well despite the fact that you can’t think clearly now, that is strange. And you can rarely make up your mind, now this does not make sense. I mean how can I only think clearly some of the time and I can't make my mind up about anything, but I can deal with problems well often!” (Female, 67)

#### Non-linear effects of levels

Each of the five attribute levels influenced differently to participants’ overall impression of a state. As mentioned by two participants, unit changes in attribute levels were not equally valued.

“It's like a sort of a diminishing return... when you go from none of the time to rarely, it is a big jump. But then rarely to some of the time is still quite a big jump. Then some of the time to often is a smaller jump. Then from often to all of the time... it reduces....?” (Male, 32)

#### Inferiority of top levels

Although FMWB is theoretically feasible, one participant rejected the idea of perfection in MWB and preferred a dominated alternative without “all of the time” for all seven items (i.e. non-monotonic valuation). The justification was that a maximal well-being state represented a lack of challenging life experience, which was a crucial element of an exciting and balanced life. Also, FMWB was considered unrealistic and could imply a lack of awareness or illusionary thinking, the failure to recognise individuals’ self-position.

“I really struggled with... the whole concept of FMWB, because FMWB as described... is too perfect. I don't believe it and I don't like it... I'm a human being, I have ups and downs, that's quite normal and healthy. And it would be really unhealthy to be in this perfect state of MWB all of the time because... what's life about?” (Female, 67)

### Theme 3: decision heuristics

Various decision strategies were found during the C-TTO and DCE valuation process.

#### Lexicographic ordering

Participants normally put more weight on important items and less for relatively unimportant items when interpreting the overall impression of a state. However, six participants exhibited a non-compensatory preference, in which they selected a preferred option based on a subset of the most important attribute(s) [38]. This violation of the continuity axiom was particularly obvious in the completion of the DCE exercise as they failed to trade off all attributes when making a final decision.

“They might instinctively [be] going towards option B… just because you're relaxed, you've got people close to you…” (Male, 32)

#### Interpretation of levels

Nine participants considered the existence of extreme levels at the highest end and the lowest end of the response category. They preferred a state with more balanced attribute levels, which were considered preferable for achieving multiple aspects of MWB.

“I would go for B because I think A seems more extreme like none none, and then all all, whereas B is... you know only got one all and one none. So it's sort of more middle of the road”. (Female, 29)

Four participants chose a preferred state with a higher level-sum score by counting the number of occurrences of each level in a state.

#### Personal and external factors

Participants with different demographic background (e.g. ages and occupations), personal judgements and characteristics (e.g. habits, outlook and commitments in life) influenced preferences towards MWB states. Furthermore, the existence of external support would increase the acceptability of a particular state.

“Possibly I don't make up my mind about things, I'll leave things to her (i.e. his wife)…” (Male, 28)

#### Availability heuristic

Eight participants assessed the frequency of a class or the probability of an event by the ease with which instances could be brought to mind [39]. They explained their impression of a state by recalling daily examples (e.g. news reports and relatives’ experiences) and past experiences.

“I have a brother-in-law... … who had a stroke when he was... late forties… … so I think this might kind of almost describe him. Because emotively he's still there, but physically... he’s not... able to do anything and mentally, he's not able to be doing... very much.” (Female, 67)

Four participants used an analogy to illustrate the meaning of a state.

“Not able to make up your own mind at all...... again that’s a bit like... being in a prison or institutionalised or something if you can't ever make any decisions for yourself...” (Male, 32)

#### Rejection of unimaginable states

One participant observed that their decision to select a particular state within a DCE pair was sometimes informed by the elimination of an unimaginable state.

“Sometimes I was choosing the other one, not necessarily because I preferred it, but because I rejected one. It's like I just don't believe that.” (Female, 67)

#### Theme 4: valuation feasibility

Difficulties such as imagination of states and quantification of years in the C-TTO tasks accentuated cognitive burden. Some participants also felt overwhelmed when completing forced DCE pairs as the process of comparing alternative permutations of levels for seven attributes induced information fatigue.

“It was tough... but... doable... in terms of... used quite a brainpower... it's just you're trying to hold a lot of things in your mind at the same time as you've got the profile of attributes on the left and then the profile on the right, and then is just trying to weight those up simultaneously.” (Male, 32)

However, all participants found the interviews manageable and the C-TTO and DCE tasks complementary. Participants also acknowledged the importance of the C-TTO practice tasks to relieve uncertainties from mere description of instructions and recognise their standard and position on time preference. The C-TTO and DCE tasks were beneficial and allowed them to reflect on life and their personal preferences.

### Theme 5: valuation outcome

#### Failure to reach the C-TTO indifference point

One participant with prior experience of mental illness failed to reach the indifference point for four states even after exhausting all lead time in the worse-than-death scenario. Particularly, for the lowest state 1,111,111, she found it distressing and was not willing to live in this state, no matter how many years of lead time were given ahead of it. This constituted the value of -∞. Among those participants who valued states as better than death, one participant failed to reach the indifference point for some states due to her dislike of the concept of FMWB. The task failed to proceed as it violated the theoretical assumption of setting FMWB as the best state. This implied a value of > 1.

#### Non-trading effects

Nine participants were not willing to give up years of life if the states were considered sufficiently promising.

“I would be happy with either of those, because none of them are particularly... gonna make you sad, are they? Okay, it’s not... all of the time, but I don’t think life in general is like that... ...” (Female, 51)

## Discussion

This paper summarises the issues identified through the cognitive process of completing C-TTO and DCE tasks for the valuation of the SWEMWBS. Implications for modifications (Table 6) and other interview findings are discussed in this section.

**Table 6:** Issues identified by the interview and the corresponding proposed modification to the valuation protocol

Issue identified	Related section	Proposed modification
Inappropriate C-TTO practice examples	3.1.1	One additional version of practice example related to physical health and relationship
Confusion about the time trade-off procedure	3.1.2	More detailed explanations of the instructions Slowing the instructing speed Encouraging participants to raise questions Clarification of practice states before completion More step-by-step trade-off demonstrations
Visual difficulty in differentiating the states within the C-TTO feedback module	3.1.3	Guidance to enhance the readability of the states line-by-line will be provided
Incomprehensible combinations of levels of attribute	3.2.1	The selection of experimental design choice sets with potential uncommonly reported states could be avoided
The exhibition of lexicographic ordering	3.3.1	Participants will be instructed to consider all attributes within the allocated states
The existence of preference heterogeneity	3.3.3	Advanced modelling techniques with the inclusion of covariates and interaction terms could be applied
Visualisation of states from a third party perspective	3.3.4	Participants will be told by the instruction to imagine themselves being in the allocated states
Promising manageability of the number of tasks	3.4	The number of tasks for each of the C-TTO and DCE parts will be increased from 8 to 10 (i.e. 10 C-TTO and 10 DCE tasks)

*C-TTO* indicates composite time trade-off, *DCE* discrete choice experiment

First, the style and structure of the C-TTO questions were challenged. The cognitive burden from imagination for the practice questions identified in this study was not documented in the wheelchair examples used in the EQ-5D-5L valuation studies [40]. This could be explained by the generic nature of physical health issues, as these were applicable to people with different ages. Considering this, one additional version of generic practice example related to physical health and relationships (Online Resource 2) was added to the original versions of the job application and relationship examples in the follow-on SWEMWBS valuation study. Participants are given the flexibility to choose between two practice versions. Also, inexperienced participants unintentionally made mistakes even after practising because of the complexity of the C-TTO completion. The presentation context was improved by deepening and slowing the instruction explanations. Clarification of the meaning of the life A and life B scenarios after each move are now described, ensuring that participants recognise the trade-off purpose. To enhance the visual readability of the C-TTO FM, more guidance on reading the pooled states line-by-line is now provided. This slide was useful to check the robustness of the results as more than half of the participants flagged problematic rank ordering of states. The modelling results from other country-specific EQ-5D-5L value sets with the adoption of EQ-VT also showed a goodness-of-fit improvement after dropping flagged states [41, 42]. Some participants suggested corrections to rank ordering deliberately by allowing swapping between states. However, arguably, this would sacrifice the role of C-TTO in deriving the value of states. To keep the C-TTO theoretical foundation, with reference to the EQ-VT, data from those flagged invalid states will be deleted and no swapping of states will be required after indicating disagreements [31].

Some features of the valuation items and levels were identified by the interviews. Regarding potential conflicting combination of levels of attributes within a state, national datasets in the U.K. that include the SWEMWBS were separately analysed to explore characteristics of response patterns to the measure (Online Resource 3). Interestingly, there was insufficient evidence to exclude any SWEMWBS states, as the implausible states claimed by participants were not uncommon in national survey responses. Instead of state exclusion, when allocating choice sets to participants, the selection of experimental designs with potential uncommonly reported states can be avoided among many iterations. Moreover, regarding the non-linear effects of attribute levels, dummy coding was used in the utility specification of the DCE experimental design [43]. The interview results supported this assumption, as some participants indicated that they placed different weights on different levels. Lastly, no valid conclusion about the issue of non-monotonic valuation (i.e. not preferring FMWB) on the suitability of C-TTO technique can be made as this was only identified by one participant.

Additionally, completion heuristics were discovered. The participants’ weighting of items was affected by the framing of the tasks (i.e. C-TTO-matching tasks versus choice-based DCE tasks) and the combination of levels of attributes. The presence of lexicographic ordering, a focusing effect normally discovered when respondents interpreted a state [26], caused the failure to reflect full preference when some attributes were unattended. Moreover, the strategy of solely interpreting the level-sum score of states within the DCE pairs posed a risk of neglecting the essence of items. Considering these, participants should be reminded to interpret a state with both its levels and attributes before task completions. This could encourage them to take as much information into account as possible, even though not every attribute was equally important for them. Furthermore, values attached to a specific state were influenced by the variation in individuals’ characteristics and tastes (i.e. preference heterogeneity). Choice models can explain deterministic (across observed individual characteristics) and random (unobserved) heterogeneities [44]. Furthermore, a few participants visualised states through the lens of available examples in society or through a third-party state. Participants are now reminded that the theoretical setting of both C-TTO and DCE techniques requires them to primarily immerse *themselves* into the allocated scenarios rather than imagining how others would behave in the state.

Concerning the manageability of the exercise, both C-TTO and DCE exercises are being maintained for the follow-on valuation study to allow different aspects of analysing preferences. Even though some participants felt cognitively exhausted to answer a forced DCE pair, the idea of a forced choice was to maximise the trade-offs between items and avoid the loss of power [45]. It could sometimes be difficult for participants to compare alternative permutations of levels for seven attributes. However, it was considered impossible to further reduce the number of items as the SWEMWBS descriptive system has already undergone comprehensive Rasch analyses [46, 47]. Keeping several levels of items identical between the two DCE alternatives could have relieved participants’ cognitive burden. However, as documented in other studies which tested the effect of overlapping some dimensions across pairs [48], participant’s neglect of these identical items made the trade-off decision less informative. As all participants found the number of tasks within the interview manageable and a majority expressed the ability to complete more tasks, the number of tasks for both the C-TTO and DCE in the follow-on valuation study is increased by two each.

Finally, regarding the valuation outcome, there was only one participant who failed to reach the indifference point for some C-TTO tasks. A decision on the need to extend the amount of lead time will be investigated in the larger valuation study. The issue of non-trading could be a potential limitation for the adoption of the C-TTO technique for the valuation of SWEMWBS due to the lack of discriminatory potential. The distribution of the derived C-TTO values will be investigated in the results of the larger valuation study, to discover any potential clustering of the values at 1.

The limitations of this study include its small sample size. This study was conducted as the COVID-19 pandemic was unfolding, which restricted and ultimately curtailed our ability to identify participants for face-to-face interviews. The preference data collected were highly limited to individuals within an academic environment, even though effort was exerted to include non-academic staff. The predominance of university staff or students (ten participants) in the sample may have influenced the results. The fact that data saturation was reached suggests that the study was able to identify the main issues in spite of these limitations, but there was insufficient data to assess whether the issues raised by one participant were of broader concern. Moreover, the valuation tasks were randomly allocated to participants, without tailoring tasks consistently for each participant to test the potential violation of axioms of utility theory in their responses.

## Conclusions

This study constitutes the first attempt to apply health state valuation techniques to the valuation of MWB as measured by the SWEMWBS. The results from the cognitive interviews support the feasibility of this application and provides insights that inform the optimisation of the valuation protocol.

## Supplementary Information

Below is the link to the electronic supplementary material.

Supplementary file1 (PDF 81 kb)

Supplementary file2 (PDF 48 kb)

Supplementary file3 (PDF 134 kb)
