# Supplementary material for: The use of composite time trade-off and discrete choice experiment methods for the valuation of the Short Warwick-Edinburgh Mental Well-being Scale (SWEMWBS): a think-aloud study
Source: Qual Life Res. 2022 Mar 24;31(9):2739–51. doi: 10.1007/s11136-022-03123-0 (PMC8942805; doi:10.1007/s11136-022-03123-0)
Supplement: Supplementary file 1 — Supplementary file1 (PDF 81 kb) [file 11136_2022_3123_MOESM1_ESM.pdf]

Supplementary Information – ESM_1

Article title: The use of composite time trade-off and discrete choice experiment methods for the valuation of the Short Warwick-Edinburgh Mental Wellbeing Scale (SWEMWBS): A think-aloud study

Journal name: Quality of Life Research

Author names: Hei Hang Edmund Yiu[1], Hareth Al-Janabi[2], Sarah Stewart-Brown[3], Stavros Petrou[4], Jason Madan[5]

[1,5] Clinical Trials Unit, Warwick Medical School, University of Warwick, Coventry, United Kingdom

[2] Institute of Applied Health Research, College of Medical and Dental Sciences, University of Birmingham, Edgbaston, Birmingham, United Kingdom

[3] Division of Health Sciences, Warwick Medical School, University of Warwick, Coventry, United Kingdom

[4] Nuffield Department of Primary Care Health Sciences, University of Oxford, Woodstock Road, Oxford, United Kingdom

Corresponding author: Hei Hang Edmund Yiu, Clinical Trials Unit, Warwick Medical School, University of Warwick, Coventry, United Kingdom

E-mail address of the corresponding author: H.Yiu@warwick.ac.uk

Fig. 1 The SWEMWBS descriptive system

Below are some statements about feelings and thoughts.

Please tick the box that best describes your experience of each over the last 2 weeks

STATEMENTS	None of the time	Rarely	Some of the time	Often	All of the time
I've been feeling optimistic about the future	1	2	3	4	5
I've been feeling useful	1	2	3	4	5
I've been feeling relaxed	1	2	3	4	5
I've been dealing with problems well	1	2	3	4	5
I've been thinking clearly	1	2	3	4	5
I've been feeling close to other people	1	2	3	4	5
I've been able to make up my own mind about things	1	2	3	4	5

"Short Warwick Edinburgh Mental Well-Being Scale (SWEMWBS)
© NHS Health Scotland, University of Warwick and University of Edinburgh, 2008, all rights reserved."
