# Supplementary material for: The use of composite time trade-off and discrete choice experiment methods for the valuation of the Short Warwick-Edinburgh Mental Well-being Scale (SWEMWBS): a think-aloud study
Source: Qual Life Res. 2022 Mar 24;31(9):2739–51. doi: 10.1007/s11136-022-03123-0 (PMC8942805; doi:10.1007/s11136-022-03123-0)
Supplement: Supplementary file 2 — Supplementary file2 (PDF 48 kb) [file 11136_2022_3123_MOESM2_ESM.pdf]

Supplementary Information – ESM_2

Article title: The use of composite time trade-off and discrete choice experiment methods for the valuation of the Short Warwick-Edinburgh Mental Wellbeing Scale (SWEMWBS): A think-aloud study

Journal name: Quality of Life Research

Author names: Hei Hang Edmund Yiu[1], Hareth Al-Janabi[2], Sarah Stewart-Brown[3], Stavros Petrou[4], Jason Madan[5]

[1,5] Clinical Trials Unit, Warwick Medical School, University of Warwick, Coventry, United Kingdom

[2] Institute of Applied Health Research, College of Medical and Dental Sciences, University of Birmingham, Edgbaston, Birmingham, United Kingdom

[3] Division of Health Sciences, Warwick Medical School, University of Warwick, Coventry, United Kingdom

[4] Nuffield Department of Primary Care Health Sciences, University of Oxford, Woodstock Road, Oxford, United Kingdom

Corresponding author: Hei Hang Edmund Yiu, Clinical Trials Unit, Warwick Medical School, University of Warwick, Coventry, United Kingdom

E-mail address of the corresponding author: H.Yiu@warwick.ac.uk

Table 1 An additional version of C-TTO practice example

**Designated mental well-being state**	You have just been for a check up with your local GP surgery who has told you that you have somewhat raised blood pressure and possible signs of diabetes. The surgery has now just telephoned to say that your blood tests have shown that a very high cholesterol and confirmed a diagnosis of diabetes. As a result, you will have to start taking pills to reduce your risk of heart disease and control your blood sugar, and also go onto a special diabetic diet. Your results mean you are at higher risk of heart disease in the future than the general population. As a result, you are very worried about your health and how you are going to manage your new diet.
**A mental well-being state which is much higher than the designated mental well-being state**	Now imagine that the surgery just contacted you to say that your blood tests for cholesterol and blood sugar levels are normal so you do not have to take any pills, and your risk of heart diseases is typical for a healthy person of your age. As a result, you feel greatly relieved and both happy and relaxed.
**A mental well-being state which is much lower than the designated mental well-being state**	Now imagine that the results from your check up showed you had diabetes and very high cholesterol and also that you have had to move house recently and have lost contact with your close friends. You feel very worried about your health, and also feel lonely and isolated because you have no-one to talk through your problems with, or to do activities you enjoy which might take your mind off your problems.
