# Supplementary material for: The use of composite time trade-off and discrete choice experiment methods for the valuation of the Short Warwick-Edinburgh Mental Well-being Scale (SWEMWBS): a think-aloud study
Source: Qual Life Res. 2022 Mar 24;31(9):2739–51. doi: 10.1007/s11136-022-03123-0 (PMC8942805; doi:10.1007/s11136-022-03123-0)
Supplement: Supplementary file 3 — Supplementary file3 (PDF 134 kb) [file 11136_2022_3123_MOESM3_ESM.pdf]

Supplementary Information – ESM_3

Article title: The use of composite time trade-off and discrete choice experiment methods for the valuation of the Short Warwick-Edinburgh Mental Wellbeing Scale (SWEMWBS): A think-aloud study

Journal name: Quality of Life Research

Author names: Hei Hang Edmund Yiu[1], Hareth Al-Janabi[2], Sarah Stewart-Brown[3], Stavros Petrou[4], Jason Madan[5]

[1,5] Clinical Trials Unit, Warwick Medical School, University of Warwick, Coventry, United Kingdom

[2] Institute of Applied Health Research, College of Medical and Dental Sciences, University of Birmingham, Edgbaston, Birmingham, United Kingdom

[3] Division of Health Sciences, Warwick Medical School, University of Warwick, Coventry, United Kingdom

[4] Nuffield Department of Primary Care Health Sciences, University of Oxford, Woodstock Road, Oxford, United Kingdom

Corresponding author: Hei Hang Edmund Yiu, Clinical Trials Unit, Warwick Medical School, University of Warwick, Coventry, United Kingdom

E-mail address of the corresponding author: H.Yiu@warwick.ac.uk

An algorithm to explore potential highly uncommon reported SWEMWBS states

It was acknowledged that there are two datasets with wide national SWEMWBS data. The first one was the Understanding Society (United Kingdom Household Longitudinal Study), which collected nine waves of data from members of the U.K. households between 2009 and 2018. SWEMWBS data was collected within waves 1, 4 and 7 of the main survey. After the elimination of missing and inapplicable data, there were 114,940 (Wave 1 = 38395; Wave 4 = 39062; Wave 7 = 37483) valid responses. The second one was the Health Survey for England, which collected annual national health data starting from 1991. The WEMWBS questionnaire was incorporated between 2010 and 2016 and there were 49081 valid data in total across all years (2010 = 7163; 2011 = 7196; 2012 = 5033; 2013 = 7777; 2014 = 7014; 2015 = 7897; 2016 = 7001). As not all items within the questionnaire were of interest, the responses of the 7 items of the SWEMWBS descriptive system were extracted from the WEMWBS descriptive system. The datasets from Understanding Society and Health Survey for England were then pooled together, resulting in a total number of 164021 SWEMWBS responses.

To investigate the response pattern of the SWEMWBS responses for identifying potential implausible states, the following step-by-step algorithm was proposed and applied to the 164,021 responses.

1) Identify the X most reported states.
2) For each of the 78,125 combinations of the SWEMWBS state,
   a) Calculate the sum of the absolute level distances across attributes between the state and each of the X states identified in step 1. For example, the sum of the absolute level distances between the state 3333333 and state 1234543 would be 2+1+0+1+2+1+0 = 7
   b) Take the minimum of the sum of the absolute level distances across attributes with each of the X states. Let this value be D_i, in which i lies between 1 and 78125.
3) Identify and exclude the Y states with the highest values for Di.

The frequency pattern and proportion among all of the 164,021 responses were computed. There were totally 12,801 reported states (frequency >0) and 65,324 states were unreported (frequency =0). A selection of the top 10 responses is reported in the Table 1 below:

Table 1 The frequency and proportion of the top 10 responses

**SWEMWBS response**	**Frequency**	**Proportion (in %)**
4444444	11161	6.80
3333333	6112	3.73
5555555	3738	2.28
3444444	2853	1.74
4434444	2806	1.71
4444445	1705	1.04
3333334	1504	0.92

3344444	1414	0.86
3434444	1402	0.85
4444434	1358	0.83

The table above shows that the most reported state was 4444444, which appeared 11,161 times and occupied 6.8% of the total number of responses. Since there was no official guidance regarding the threshold of values X and Y, X was defined as those states with the proportion greater than 0.05%. There were 301 states fitting this criteria (i.e. X = 301) and the lowest frequency of the state that met this criteria was 83 times. The sum of the absolute level distances across attributes between each of the 78,125 states and each of the 301 states were calculated and the corresponding D values were derived. The derived D values for each of the 78,125 states were ranged from 0 to 12. The higher the D value of a particular state, the larger would be its deviation from the 301 commonly reported states, and the higher would be the possibility that the state was uncommon or implausible for participants. With the application of this algorithm, Table 2 shows the D values calculated from potential implausible states claimed by participants:

Table 2 The D values of implausible states claimed by participants

**Quotes**	**Corresponding descriptive state**	**Corresponding state index**	**D value**
"This is an interesting health state. Coz it's quite sort of conflicted in terms of the very optimistic but then they don't feel useful at all and they're not relaxed so..."<br><br>*"but yeah again it's quite a difficult... it's quite a challenging task to do... just when you've got these really conflicting things... and then it's got lit with difficulty with it being..."* [Male, 32]<br><br>*"So it's almost like you're making up your mind about things, but then... but you're also rarely dealing with problems well... it's contradictory in a way... so it's like you're able to make a decision but then your decisions are wrong, if it's associated with a problem."* [Female, 35]	*often feeling optimistic about the future<br>*rarely feeling useful<br>*none of the time feeling relaxed<br>*rarely dealing with problems well<br>*some of the time thinking clearly<br>*all of the time feeling close to other people<br>*often able to make up my own mind about things	4212354	5
"Yup, okay so... rarely feeling optimistic is not a good thing, none of the time feeling useful is not good, all of the time feeling relaxed... so that feels counter-intuitive. [laugh]"<br><br>*"Er... all of the time feeling relaxed, so that feels strange to me that feeling relaxed but not being optimistic and not feeling useful, they seem to disagree with each other."*<br><br>*"I: Why do you think it is counter-*intuitive *to be none of the time feeling useful and all of the time feeling relaxed? P: Yeah... if I am that relaxed all of the time, I'd probably*	*rarely feeling optimistic about the future<br>*none of the time feeling useful<br>*all of the time feeling relaxed<br>*some of the time dealing with problems well<br>*all of the time thinking clearly<br>*often feeling close to other people<br>*all of the time able to make up my own mind about things	2153545	5

be quite optimistic about things... me personally. And if you're feeling quite relaxed... for me again, I wouldn't be worrying about whether I felt useful or not about things, I think... that's how I would feel anyway.<br>*I: So is it difficult to imagine...*<br>*P: Yes.... yeah. For me that's hard to... picture being relaxed all the time but then having these other issues going on." [Female, 43]*			
"So er... this time often feeling optimistic, often feeling useful, rarely feeling relaxed, rarely dealing with problems well, none of the time thinking clearly, none of the time feeling close to other people, some of the time being able to make up my mind about things... so... again probably going to contradict what I've just said now, but yeah feeling... rarely feeling relaxed... yeah still feels optimistic about the future, and often feeling useful." [Female, 43]	*often feeling optimistic about the future<br>*often feeling useful<br>*rarely feeling relaxed<br>*rarely dealing with problems well<br>*none of the time thinking clearly<br>*none of the time feeling close to other people<br>*some of the time able to make up my own mind about things	4422113	5
"really I don't understand how you can think clearly but not deal with problems well. If you think clearly, problems should be solved."<br><br>*"... ... You often think clearly but you... you only some of the time make up your own mind about things. So how can you be thinking clearly if you're indecisive? How can you be thinking clearly if you're unable to deal with your problems?" [Male, 32]*	*some of the time feeling optimistic about the future<br>*none of the time feeling useful<br>*often feeling relaxed<br>*some of the time dealing with problems well<br>*often thinking clearly<br>*all of the time feeling close to other people<br>*some of the time able to make up my own mind about things	3143453	4

“You know you should not feel optimistic if you never feel useful.”<br><br>*“Often feel relaxed, often deal with problems... hmm... often deal with problems well despite the fact that you can't think clearly now, that is strange. And you can rarely make up your mind, now this does not make sense. I mean how can I only think clearly some of the time and I can't make my mind up about anything, but I can deal with problems well often! This does not make sense.” [Female, 67]*	*often feeling optimistic about the future<br>*none of the time feeling useful<br>*often feeling relaxed<br>*often dealing with problems well<br>*some of the time thinking clearly<br>*often feeling close to other people<br>*rarely able to make up my own mind about things	4144342	4
“Now the other way around and I'll start with B... so I'm optimistic... I think clearly... but I don't feel useful, I don't feel relaxed, I don't think, I don't feel close to people, and I can't make my mind up, but I feel optimistic, and I think clearly, none of this makes sense. [laugh] None of this makes sense. I can think clearly and I can feel optimistic despite the fact that I don't feel useful, I don't deal with problems, I can't make my mind up and I don't feel close to people... oh this can't work.” [Female, 67]	*all of the time feeling optimistic about the future<br>*none of the time feeling useful<br>*rarely feeling relaxed<br>*none of the time dealing with problems well<br>*all of the time thinking clearly<br>*none of the time feeling close to other people<br>*none of the time able to make up my own mind about things	5121511	9
“But you might say something like I've been dealing with problems well but I can't make up my mind. And you think... well if I can't make up my mind, how can you even start to deal with problems... it just didn't make sense.” [Female, 67]	*rarely feeling optimistic about the future<br>*often feeling useful<br>*none of the time feeling relaxed<br>*often dealing with problems well<br>*rarely thinking clearly<br>*all of the time feeling close to other people<br>*rarely able to make up my own mind	2414252	7

	about things		
"... again it sounds counter-intuitive to me em... because if I'm not thinking clearly, it's... difficult to see how I'm dealing with problems well." [Male, 67]	*all of the time feeling optimistic about the future<br>*rarely feeling useful<br>*all of the time feeling relaxed<br>*often dealing with problems well<br>*none of the time thinking clearly<br>*none of the time feeling close to other people<br>*some of the time able to make up my own mind about things	5254113	8
"See again... sometimes it's rarely dealing with problems well, often thinking clearly, er... seem counter-intuitive to me. They don't... really go together." [Male, 67]	*some of the time feeling optimistic about the future<br>*often feeling useful<br>*rarely feeling relaxed<br>*rarely dealing with problems well<br>*often thinking clearly<br>*rarely feeling close to other people<br>*rarely able to make up my own mind about things	3422422	4

I indicates interviewer; P, participant.

Interestingly, the D statistics for the states which caused concerns to participants were actually not that implausible since they were not that dissimilar from states actually reported in the national survey data. Most of the D statistics were around 5, with only one state with D-statistics of 9. In this context, there was insufficient evidence to rule out any states when running the experimental designs of C-TTO and DCE. However, it was worth noting that the selection of those choice sets which include the states with D-values of 10 or above could be avoided, with a view to minimise imagination burden. It should be stressed that we are not suggesting the deletion of those highly uncommon states as they will still be extrapolated during the modelling of utility values when conducting a larger SWEMWBS valuation study.
